# Supplementary material for: Fast photothermal spatial light modulation for quantitative phase imaging at the nanoscale
Source: Nat Commun. 2021 May 19;12:2921. doi: 10.1038/s41467-021-23252-3 (PMC8134576; doi:10.1038/s41467-021-23252-3)
Supplement: Supplementary file 1 — Supplementary Information [file 41467_2021_23252_MOESM1_ESM.pdf]

Supplementary Information for

Fast photothermal spatial light modulation for quantitative phase imaging at the nanoscale

Hadrien M.L. Robert[1], Kristýna Holanová[1], Łukasz Bujak[1], Milan Vala[1], Verena Henrichs[2], Zdeněk Lánský[2] and Marek Piliarik[1,]*

[1]Institute of Photonics and Electronics of the Czech Academy of Sciences, Chaberská 1014/57, 18251 Prague, Czech Republic.

[2]Institute of Biotechnology of the Czech Academy of Sciences, BIOCEV, Průmyslová 595, 252 50 Vestec, Czech Republic

E-mail: piliarik@ufe.cz,

Content:

Supplementary Note 1: Phase-shift induced by a 2D heat source in a 3 layers system

Supplementary Figure 1: Model of the thermo-induce phase-shift in the three-layer system

Supplementary Note 2: Effect of the superstrate material on the phase-shift profile

Supplementary Figure 2: Phase-shift profiles of a plane wave propagating through the structure

Supplementary Figure 3: Effect of the glycerol layer thickness on the phase-shift profile.

Supplementary Figure 4: Heating sample characteristic

Supplementary Note 3: Characterization of the thermo-induced wavefront deformation

Supplementary Figure 5: Characterization of the thermo-induced wavefront deformation

Supplementary Figure 6: Experimental phase-shift characterization.

Supplementary Figure 7: Localization precision under heating modulation.

Supplementary Note 4: Amplitude and phase estimation

Supplementary Figure 8: Amplitude and phase estimation

Supplementary Note 5: 3D images reconstruction

Supplementary Figure 9: 3D images reconstruction

Supplementary Note 6: Single microtubule on kinesin-coated surfaces

Supplementary Figure 10: Effect of the kinesin-1 concentration on the microtubule height profile.

Supplementary Figure 11: The microtubule crosslinker PRC1 switches between microtubules.

Supplementary References

Supplementary Note 1: Phase-shift induced by a 2D heat source in a three layers system

Here we describe the theory of the thermo-induced phase shift and our simulation parameters.

Single medium and point heat source case

If we consider a point heat source delivering a power Q in a homogeneous liquid medium, the temperature distribution $T(r)$ in steady-state is governed by the following heat equation:

$$\nabla^2 T(r) + \frac{Q}{\kappa}\delta(r) = 0, \qquad (1)$$

where ∇^2 is the Laplacian operator, κ is the thermal conductivity of the medium (W.K^{-1}.m^{-1}), and r is the distance from the heat source (m) in a spherical coordinate system. A solution of the heat equation yields:

$$T(r) = \frac{Q}{4\pi\kappa r} + T_\infty, \qquad (2)$$

$$T(r) = QG(r) + T_\infty, \qquad (3)$$

where T_∞ is the temperature far from the heat source, and $G(r) = 1/(4\pi\kappa r)$ is the Green's function of the heat equation.

Green's function for a three-layer system

The structure used in our experiments comprises three layers: a 20 µm thick liquid glycerol sandwiched between a glass coverslip and a sapphire window, both of a thickness of 140 µm. For a three layers system, the Green's function takes a more complex form.[1] The schematic Supplementary Figure 1a summarizes the considered system. We identify κ_1 , κ_2, κ_3 as the thermal conductivity coefficients of glass, glycerol, and sapphire, respectively and Δ_1 , Δ_2 , Δ_3, their respective thickness. The calculation is carried out in a cylindrical coordinate system (ρ, z) with ρ, the 2D radial distance from the heat source, and the z, axial coordinate from the heat source. The heat source takes a form of a disk. To calculate Green's function, the point heat source is located at the position ρ= 0 and z = 0. The Green's function $G(\rho, z)$ in this geometry was previously derived within the three respective layers as:

Medium 1

$$-\Delta_1 < z \leq 0$$

$$G(\rho, z) = \int_0^\infty B \frac{J_0(h\rho)}{2\pi\, A} \mathrm{d}h, \tag{4}$$

where

$$B = (\kappa_2 + \kappa_3)\exp(hz) + (\kappa_2 - \kappa_3)\exp[-h(2\Delta_2 - z)], \tag{5}$$

$$A = (\kappa_2 + \kappa_3)(\kappa_2 + \kappa_1) - (\kappa_2 - \kappa_1)(\kappa_3 - \kappa_2)\exp(2h\Delta_2)\ , \tag{6}$$

J_0 is the Bessel function of the 0th order and h the parameter used for the integration.

Medium 2

$$0 < z \leq \Delta_2$$

$$G(\rho, z) = \frac{1}{4\pi R\kappa_2} + \int_0^\infty C \frac{J_0(h\rho)}{4\pi\kappa_2\, A} \mathrm{d}h, \quad (7)$$

where

$$R = \sqrt{\rho^2 + z^2}$$

and

$$C = (\kappa_2 - \kappa_3)(\kappa_2 - \kappa_1)\exp[-h(2\Delta_2 - z)] + (\kappa_2 - \kappa_3)(\kappa_2 + \kappa_1)\exp[-h(2\Delta_2 - z)]$$
$$+ (\kappa_2 - \kappa_1)(\kappa_2 + \kappa_3)\exp(-hz) + (\kappa_2 - \kappa_3)(\kappa_2 - \kappa_1)\exp[-h(2\Delta_2 + z)].$$

Medium 3

$$\Delta_2 < z \leq \Delta_3$$

$$G(\rho, z) = \int_0^\infty D \frac{J_0(h\rho)}{2\pi\, A} \mathrm{d}h, \quad (8)$$

Where

$$D = (\kappa_2 + \kappa_1)\exp(hz) + (\kappa_2 + \kappa_1)\exp(-hz).$$

2D heat source in the three-layered structure

To analyze the temperature distribution of a 2D heat source, we define the 2D profile of a symmetrical heat source density $Q(\rho)$ (Wm^2). The temperature is calculated by a convolution product between $Q(\rho)$ and $G(\rho, z)$:

$$T(\rho, z) = Q(\rho) \otimes G(\rho, z) + T_{\infty.} \quad (9)$$

Thermo-induced refractive index changes and optical-path difference

The refractive index change associated with the temperature is expressed as $\Delta n_i(\rho, z) = n_i\big(T(\rho, z)\big) - n_i(T_\infty)$ and the dependence of refractive index on the temperature $T(\rho, z)$ can be estimated by the Taylor series:

$$\Delta n_i(\rho, z) = \sum_{j=1}^{M} b_{i,j} T^j(\rho, z), \quad (10)$$

where n_i is the refractive index of the medium i and $b_{i,j}$ represents empirical coefficients depending on the material of the medium i. If we consider an incoming light beam of a wavelength λ, propagating in the direction of the *z*-axis, the thermo-induced phase-shift φ_{therm} reads:

$$\varphi_{therm}(\rho) = \frac{2\pi}{\lambda}\left(\int_{-\Delta_1}^{0} \Delta n_1(\rho, z)\mathrm{d}z + \int_{0}^{\Delta_2} \Delta n_2(\rho, z)\mathrm{d}z + \int_{\Delta_2}^{\Delta_3} \Delta n_3(\rho, z)\mathrm{d}z\right). \quad (11)$$

Simulation parameters

In numerical simulations showed in Figure 1, we considered a disk heat source with a diameter of 60 µm, delivering heating power ranging from 0.4 to 14 µWµm^{-1}, a glycerol thickness *L*=5, 20 and 250 µm with a thermal conductivity κ=0.285 Wm^{-1}K^{-1}, a 140 µm thick glass BK7 layer with κ =1.2 Wm^{-1}K^{-1} and a 140 µm sapphire layer with κ =27.21 Wm^{-1}K^{-1}. To calculate the refractive index variation of glycerol, we use the first order of Taylor expansion coefficient: for glycerol $\Delta n/\Delta T$ =-2.7 × 10-4 K^{-1}, for BK7 glass $\Delta n/\Delta T$ = 3 × 10^{-6} K^{-1} and sapphire $\Delta n/\Delta T$ =13 × 10^{-6} K^{-1}. Simulations were processed with the Matlab software (R2018a). Supplementary Figure 1.b-j shows all the steps to obtain the phase profile with a glycerol thickness of 20 µm.

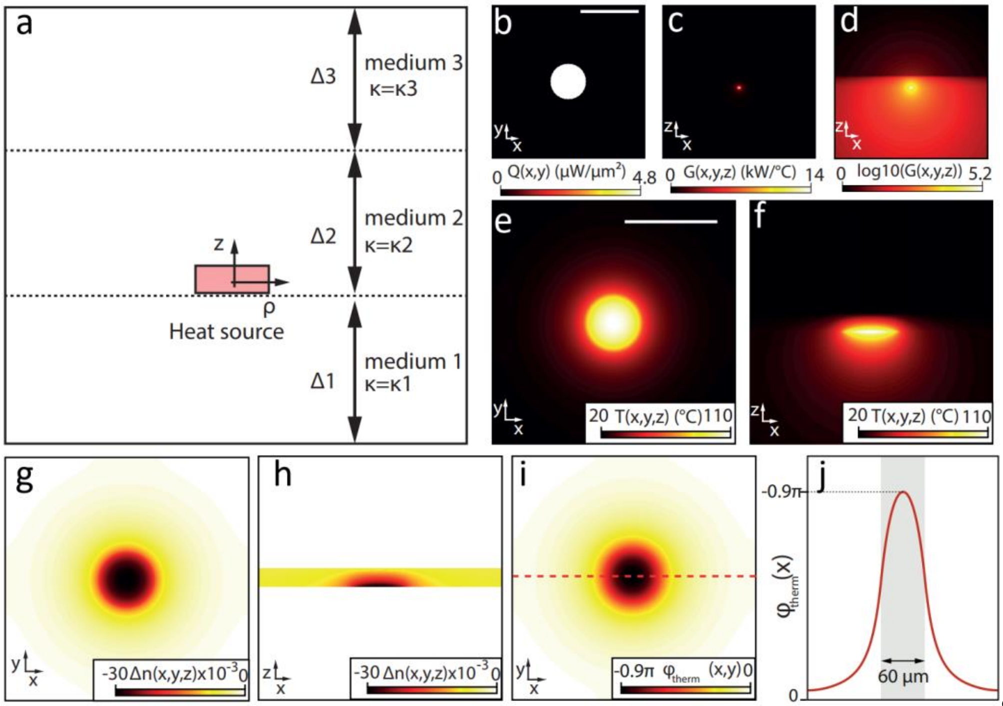

a

Supplementary Figure 1. Model of the thermo-induce phase-shift in the three-layer system: 140 µm glass, 20 µm glycerol, 140 µm sapphire. a) Schematic representing the three-layered structure used. b) 2D map of the heat source $Q(\mathrm{x,y})$; heating power of 4.8 µWµm^2; scale bar indicates 100 µm. c) 2D map of the Green's function G(x,y,z) in the *xz* cross-section. d) Logarithmic scale of G(x,y,z). e) 2D map of temperature T(x,y,z) in the *xy* cross-section and f) *xz* cross-section. g) 2D map of refractive index change Δn(x,y,z) in the *xy* cross-section and h) *xz* cross-section. i) 2D map of the phase-shift $\varphi_{therm}(x,y)$ of a plane wave propagating through the structure in *z* (λ=488 nm). j) Phase-shift profile at the position of the red dashed lines in i).

Supplementary Note 2: Effect of the superstrate material on the phase-shift profile

Supplementary Figure 2 depicts the comparison of the phase-shift profile for sapphire and BK7 glass superstrate. Sapphire has a thermal conductivity about 30 times higher than BK7 glass and acts as a heat sink resulting in better confinement of the temperature change around the heat source (here 60 µm in diameter).

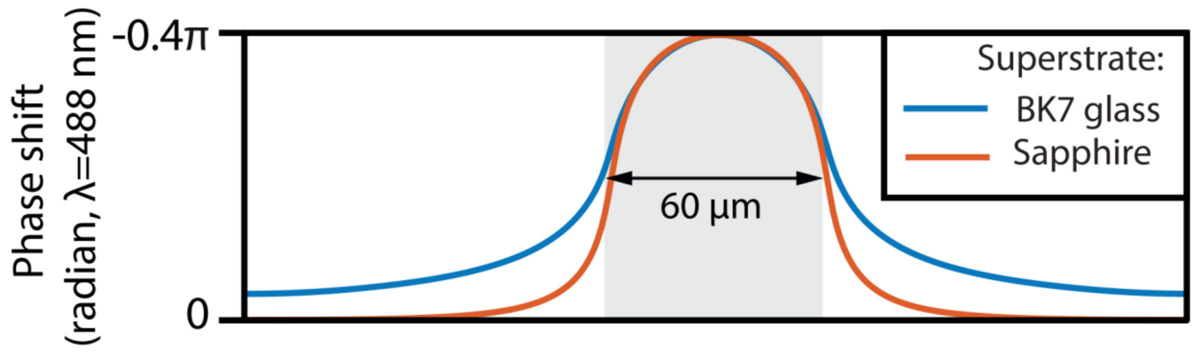

Supplementary Figure 2. **Phase-shift profiles of a plane wave propagating through the structure.** Layered structure comprising: a glass BK7 substrate (140 µm thick), a glycerol layer (5 µm thick), and two different materials of the superstrate (sapphire and BK7 glass, both 140 µm thick). A wavelength of λ=488 nm was used. To achieve comparable phase-shift amplitude, the heating power of 6.5 µWµm^2 was used in the case of the BK7 superstrate and 14 µWµm^2 was used for the sapphire superstrate.

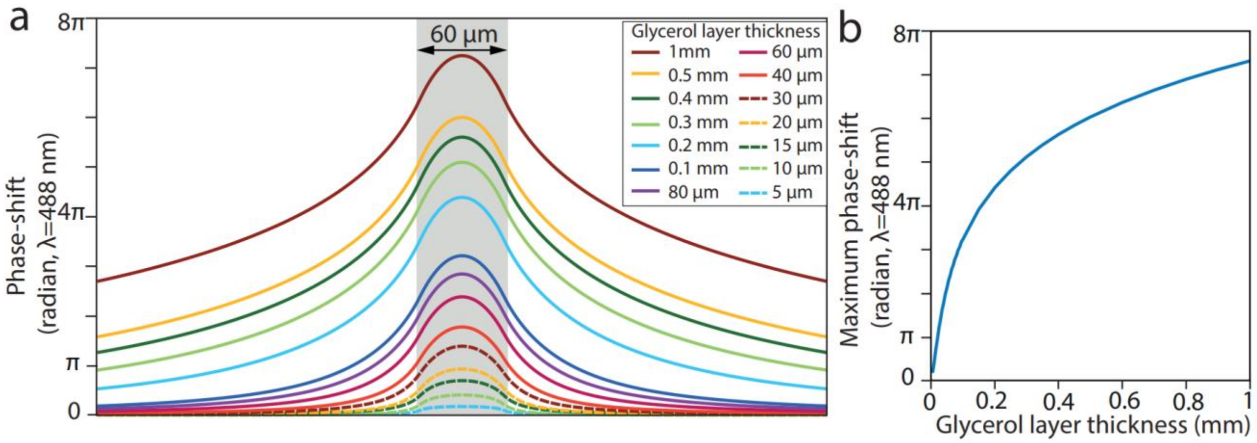

Supplementary Figure 3. **Effect of the glycerol layer thickness on the phase-shift profile.** a) The thickness of the glycerol layer ranging between 5 µm and 1 mm. Sapphire and BK7 glass were considered for layer 3 and layer 1, respectively. The 60-µm disk heat source was delivering power of 4.8 µW.µm^2. b) Dependence of the maximum phase shift as a function of the thickness of the glycerol layer.

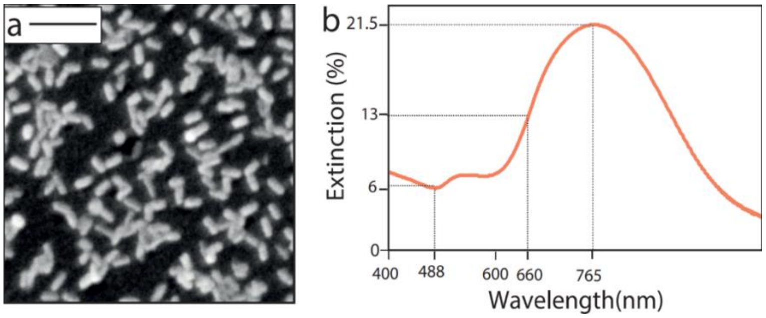

Supplementary Figure 4. Heating sample characteristic a) Scanning electronic microscopy (SEM) image of gold nanorods immobilized in the photothermal layer. (Scale bar indicates 200 nm) . b) Extinction spectrum of the layer of nanorods used for the experiments. The experiment has not been repeated.

Supplementary Note 3: Characterization of the thermo-induced wavefront deformation

To image the wavefront deformation, we employed an interferometric system similar to a DIC configuration. The PT-SLM is illuminated by a collimated light (488 nm). This light is used to image the PT-SLM on a camera with a 2.5x magnification. A Wollaston prism followed by a polarizer at 45° from the Wollaston axis is placed a few mm before the camera. Therefore, the beam is split into two shifted beams to interfere on the camera (Supplementary Figure 5a). We imaged the changes in the interference pattern due to the heating-laser illumination of a circular area of 60 µm diameter on the PT-SLM (Supplementary Figure 5b). To have a better image of the wave distortion, we subtracted the images Supplementary Figure 5b and 5a to obtain Supplementary Figure 5c. Supplementary Figure 5d shows the profile of the interference images c. We observe that the wave profile features a flat plateau of approximately 64 µm in diameter which is similar to our simulation shown in Figure 1g.

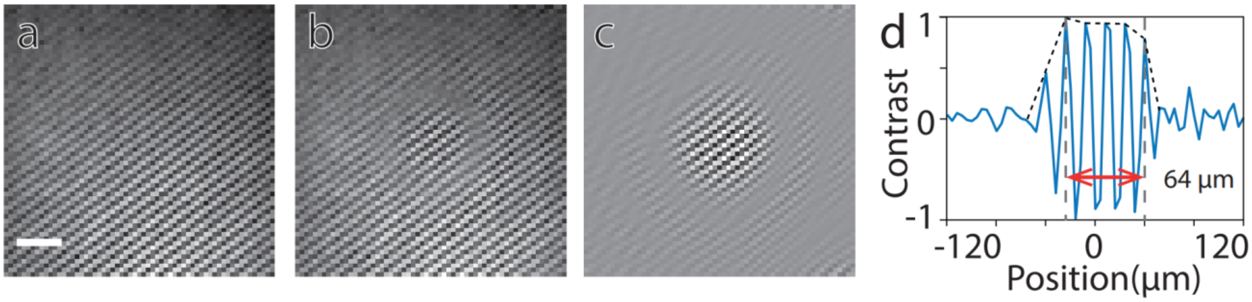

Supplementary Figure 5. Characterization of the thermo-induced wavefront deformation

a) Referenced interferometric image when the heating beam is off. Scale bar indicates 20 μm. b) Probed interferometric image when the heating beam is on. c) Subtraction of b with a. d) Linear cross-section of c. The experiment has not been repeated.

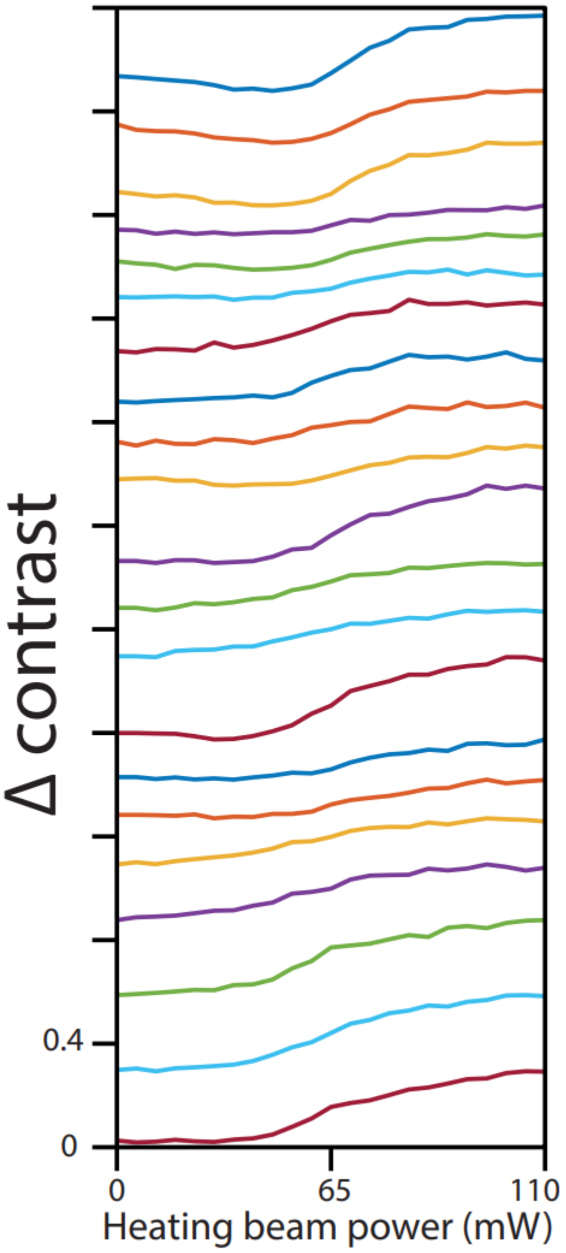

Supplementary Figure 6. Experimental phase-shift characterization. Stack of the 21 experimental curves showing the dependence of the contrast of different gold nanospheres on the heating beam power varying from 0 to 110 mW. The vertical axis represents the contrast change magnitude, and individual plots are offset for better legibility. The variation in the maximum contrast change result from the inhomogeneity in the size of nanospheres.

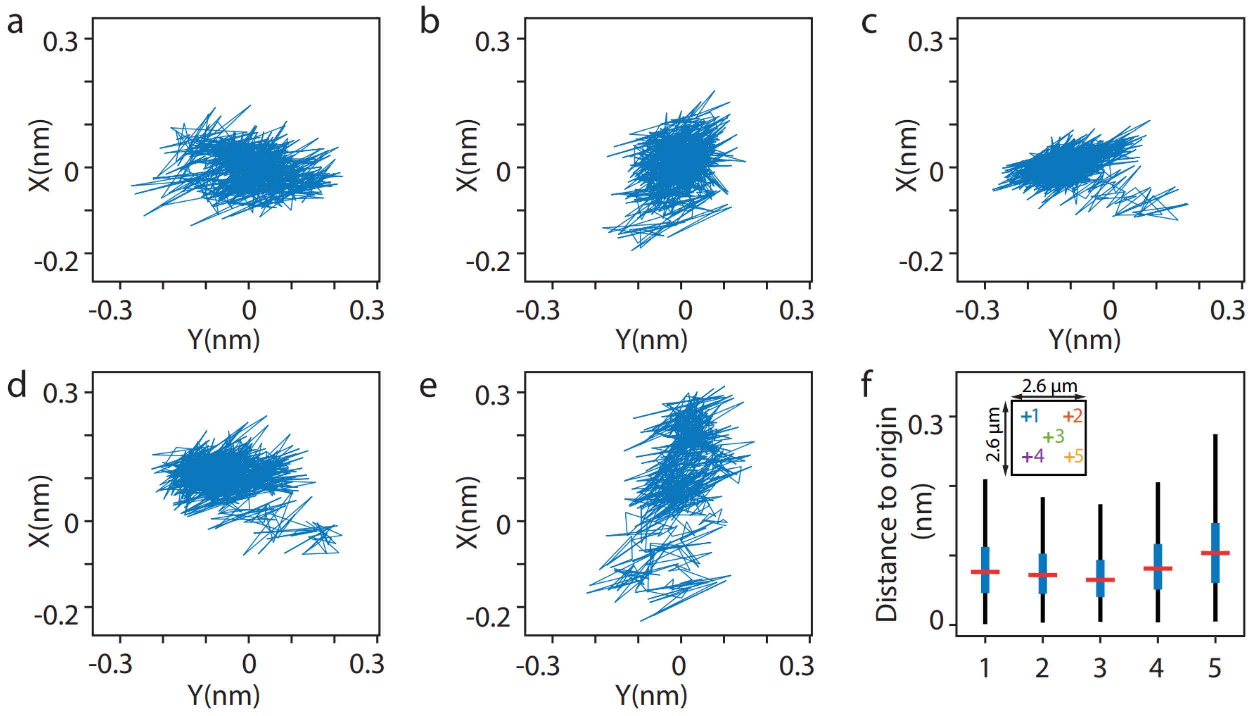

Supplementary Figure 7. Localization precision under heating modulation. a-e) 2D trace of a 20 nm gold nanoparticle respectively in 5 different positions corresponding to Figure 3e-f (shown in the inset in f). f) box plot of the distance to the origin of the trace a-e. The interquartile range is in blue, the max-min range in black, and the median in red (n=10000 successive images for each position). Inset: Field of view showing the 5 positions corresponding to the traces a-e.

Supplementary Note 4: Amplitude and phase estimation

We estimate the normalized scattering amplitude s/r and phase $\Delta\varphi$ from a single-phase sweep of the reference wave in iSCAT configuration. In principle, only four images collected at different phase shifts are sufficient to reconstruct the phase profile of the image.[2] However, with continuous modulation and sufficiently high framerate (10 000 fps) it was straightforward to obtain the full phase dependence of the contrast in a phase-shift series of 50 iSCAT images of two 30 nm gold nanospheres with a peak modulation of 1.4π showed in Supplementary Figure 7a-b. We used a least-squares fitting of the function $f(x) = A\cos\left(\frac{2\pi}{P}x + \Delta\varphi\right) + B$ on the experimental data, where P was the known period of modulation and the amplitude $A = 2(s/r)$, the phase $\Delta\varphi$ and the offset B were free-fit parameters. Supplementary Figure 6c shows the estimated intensity $(s/r)^2$ phase $\Delta\varphi$ image and corresponding correlation coefficient of the fitting. Extracting the phase and amplitude of respective Fourier components is an equivalent method in the case of perfectly linear modulation.

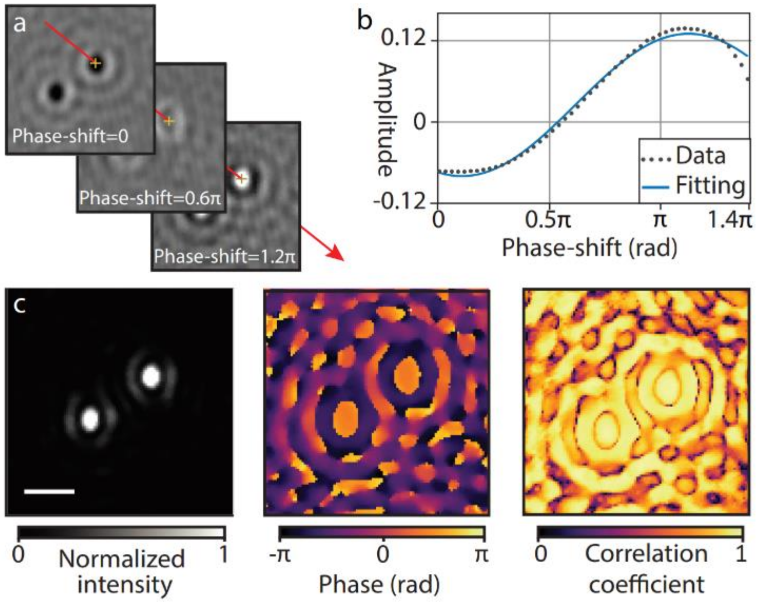

Supplementary Figure 8. Amplitude and phase estimation a) iSCAT images of two gold nanospheres (30 nm) obtained with three different phase-shift. b) Comparison of the contrast variation and the corresponding fit. c) Intensity $(s/r)^2$, phase $\Delta\varphi$ and correlation coefficient image of the two gold nanospheres. Representative images from three independent experiments, scale bar indicates 500 nm.[3]

Supplementary Note 5: 3D images reconstruction

To reconstruct the 3D profile of the crossed microtubules from iSCAT image Supplementary Figure 9a, we separated the interferences signal originating in each of the microtubules. First, we averaged the cross-section pattern in the axial direction of each microtubule. Then, we used the mean of the MT cross-section as a reference image of the single MT as shown in Supplementary Figure 9b and c. We subtracted the reference images from the image in Supplementary Figure 9a to obtain cleaned images of each microtubule as shown in Supplementary Figure 9d and Supplementary Figure 9e. 3D profiles Supplementary Figure 9f, and Supplementary Figure 9g show the profile of the phase image in Supplementary Figure 9d and Supplementary Figure 9e cropped to the diffraction-limited width of the microtubule image.

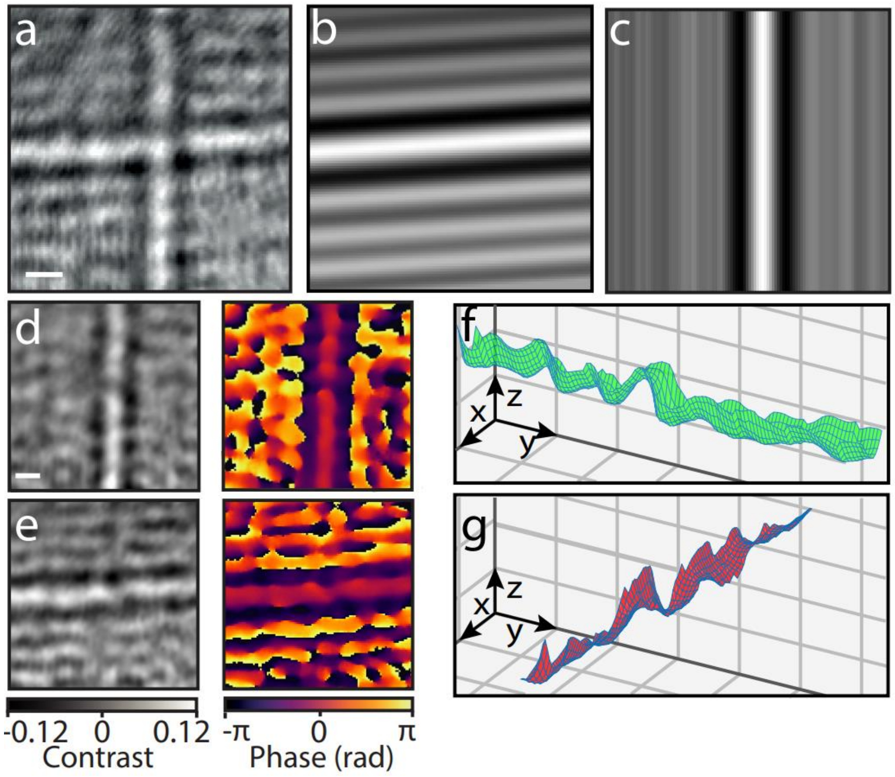

Supplementary Figure 9: 3D image reconstruction, a) iSCAT image of two overlapping microtubules. Representative images from five independent experiments, scale bar indicates 500 nm. b-c) Averaged cross-section pattern of each microtubule. d-e) separated iSCAT and phase images (from left to right) of single microtubules in a). f-g) 3D ribbons-like reconstruction calculated from the respective phase images.[3]

Supplementary Note 6: Single microtubule on kinesin-coated surfaces

We analyzed 3D images of single microtubules immobilized on a kinesin-coated surface with different functionalization densities. We incubated the surface with 1 mg/mL β-casein for 10 min. Afterward, we injected 10 µL of kinesin-1 solution for 5 min. We tested two different concentrations of the kinesin-1 of 5 $\mu g mL^{-1}$ and 25 $\mu g mL^{-1}$ in BRB80 buffer. Finally, we flushed the chamber with the attachment buffer (0.4 mM AMP-PNP (10 µL) and 10 mM DTT (10 µL) in BRB80 (980 µL) and immobilized the GDP microtubules stabilized by caps of GMCPP (a non-hydrolyzable analog of GTP) to prevent disassembly. We observed the influence of the number of kinesin-1 attachment points on the microtubule height profile in Supplementary Figure 10a-b and e-f. Interestingly with a lower number of kinesin-1 linkers, we identify considerably higher fluctuations in the microtubule height while for higher-density kinesin surface we resolve qualitatively more straight geometry of the microtubule as shown in Supplementary Figure 10(c and g) in 3D representation and Supplementary Figure 10 (d and h) as the microtubule height profile. This observation is consistent with the assumption that the flexibility of the microtubule structure depends on the number of kinesin-1 linkers immobilized on the surface. Consequently, the more kinesin-1 molecules are available at the surface, the firmer is the microtubule attachment to the surface and the straighter is its geometry.

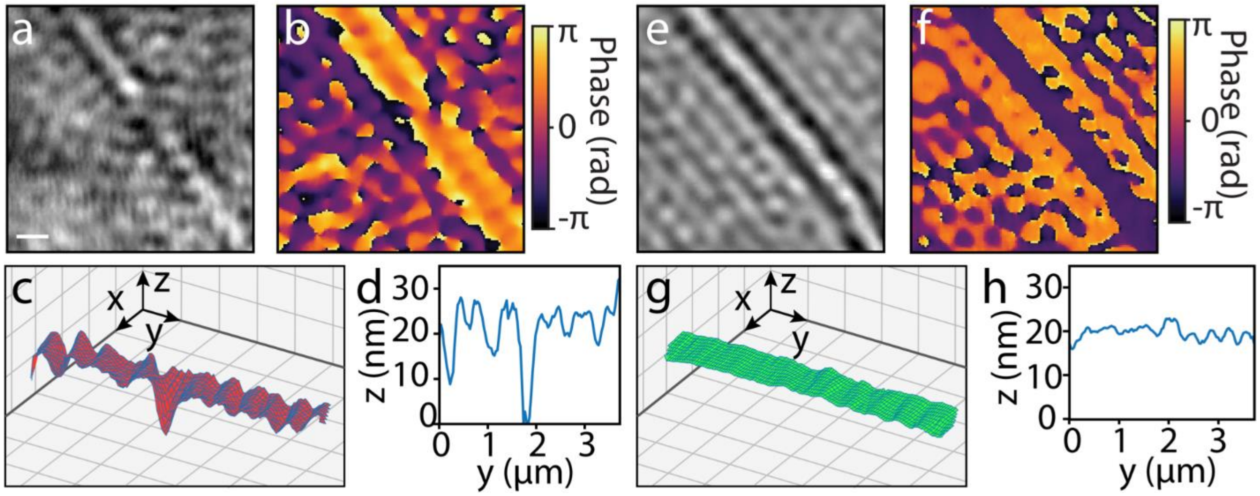

Supplementary Figure 10. Effect of the kinesin-1 concentration on the microtubule height profile. Scale bar indicates 500 nm. a) iSCAT image of a single microtubule attached to the kinesin-1 surface at 5 µgmL^{-1}. (b) Phase image corresponding to iSCAT image in a). c) 3D profile of the microtubule calculated from b). d) Height profile of the microtubule in c). e) iSCAT image of a single microtubule attached to the kinesin-1 surface at 25 µgmL^{-1}. f) Phase image corresponding to iSCAT image in e). g) 3D profile of the microtubule calculated from f). h) Height profile of the microtubule in g). The experiment has not been repeated.

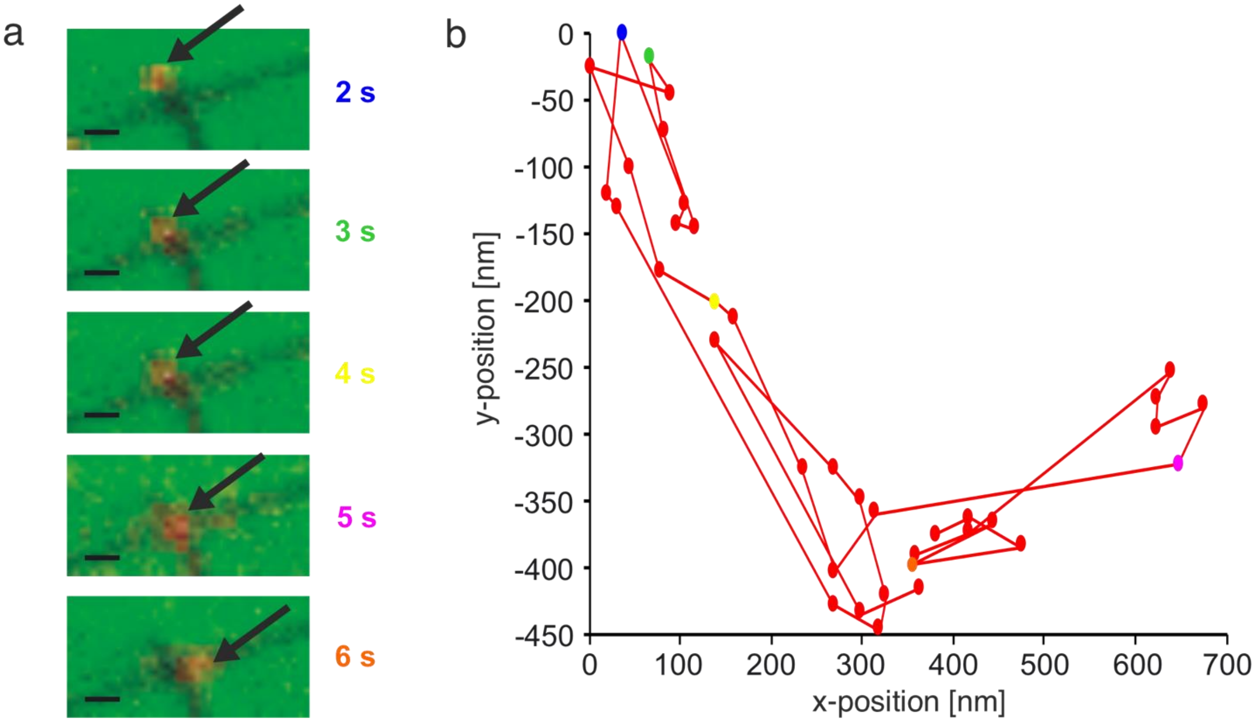

Supplementary Figure 11. The microtubule crosslinker PRC1 switches between microtubules. At a crossing of two microtubules, mRuby-PRC1 can transit from one microtubule to another by diffusion. a) Snapshots of a representative diffusive microtubule-interaction of mRuby-PRC1 (red) while switching microtubules (black) with an indication of the time point of image capture. Scale bar indicates 500 nm. b) Diagram of the trace of the diffusion of mRuby-PRC1 from subfigure (a). Each circle presents the position of mRuby-PRC1 at a particular time (frame rate 0.2 s) during imaging. Colored dots highlight the positions of mRuby-PRC1 of the respective time frames indicated in (a). The experiment has not been repeated.[3]

Suplementary references

1. Ramanan, V. S., Muthukumar, M., Gnanasekaran, S., Reddy, M. J. V. & Emmanuel, B. Green's functions for the Laplace equation in a 3-layer medium, boundary element integrals and their application to cathodic protection. *Eng. Anal. Bound. Elem.* **23**, 777–786 (1999).

2. Popescu, G. *et al.* Fourier phase microscopy for investigation of biological structures and dynamics. *Opt. Lett.* **29**, 2503–2505 (2004).

3. Robert, Hadrien; Holanová, Kristýna; Bujak, Łukasz; Vala, Milan; Henrichs, Verena; Lánský, Zdeněk; et al. (2021): Data for Fast photothermal spatial light modulation for quantitative phase imaging at the nanoscale. figshare. Dataset. https://doi.org/10.6084/m9.figshare.14170622.v3
