# Supplementary material for: Fast photothermal spatial light modulation for quantitative phase imaging at the nanoscale
Source: Nat Commun. 2021 May 19;12:2921. doi: 10.1038/s41467-021-23252-3 (PMC8134576; doi:10.1038/s41467-021-23252-3)
Supplement: Supplementary file 3 — Description of Additional Supplementary Files [file 41467_2021_23252_MOESM3_ESM.pdf]

Description of Additional Supplementary Files:

Supplementary Video 1: Reconstructed 3D trajectory of the Ase1 protein diffusion on the microtubule measured using quantitative phase imaging. The estimated envelope of the trajectory is shown with grey cylindrical lines. 1 second in the video corresponds to 36 ms in realtime. Time is color-coded on the trajectory. The last 6 points of the trajectory are highlighted in solid lines. Scale bars correspond to 20 nm
